# Iron overload suppresses hippocampal neurogenesis in adult mice: Implication for iron dysregulation‐linked neurological diseases

**DOI:** 10.1111/cns.14394

**Published:** 2023-08-07

**Authors:** Jie Li, Yiqian Ding, Jianhua Zhang, Yating Zhang, Yiduo Cui, Yi Zhang, Shiyang Chang, Yan‐Zhong Chang, Guofen Gao

**Affiliations:** ^1^ Laboratory of Molecular Iron Metabolism, Key Laboratory of Molecular and Cellular Biology of the Ministry of Education, Key Laboratory of Animal Physiology, Biochemistry and Molecular Biology of Hebei Province, College of Life Sciences Hebei Normal University Shijiazhuang China; ^2^ College of Basic Medicine Hebei Medical University Shijiazhuang China

**Keywords:** adult hippocampal neurogenesis, BDNF, furin, iron, neural stem cells, neurological diseases

## Abstract

**Aims:**

Adult hippocampal neurogenesis is an important player in brain homeostasis and its impairment participates in neurological diseases. Iron overload has emerged as an irreversible factor of brain aging, and is also closely related to degenerative disorders, including cognitive dysfunction. However, whether brain iron overload alters hippocampal neurogenesis has not been reported. We investigated the effect of elevated iron content on adult hippocampal neurogenesis and explored the underlying mechanism.

**Methods:**

Mouse models with hippocampal iron overload were generated. Neurogenesis in hippocampus and expression levels of related molecules were assessed.

**Results:**

Iron accumulation in hippocampus remarkably impaired the differentiation of neural stem cells, resulting in a significant decrease in newborn neurons. The damage was possibly attributed to iron‐induced downregulation of proprotein convertase furin and subsequently decreased maturation of brain‐derived neurotrophic factor (BDNF), thus contributing to memory decline and anxiety‐like behavior of mice. Supportively, knockdown of furin indeed suppressed hippocampal neurogenesis, while furin overexpression restored the impairment.

**Conclusion:**

These findings demonstrated that iron overload damaged hippocampal neurogenesis likely via iron–furin–BDNF pathway. This study provides new insights into potential mechanisms on iron‐induced neurotoxicity and the causes of neurogenesis injury and renders modulating iron homeostasis and furin expression as novel therapeutic strategies for treatment of neurological diseases.

## Graphical abstract

Iron accumulation in hippocampus remarkably impairs the adult hippocampal neurogenesis. This regulation is likely attributed to iron‐induced downregulation of proprotein convertase furin and the subsequent decrease in maturation of brain‐derived neurotrophic factor, which affects the differentiation of neural stem cells, thus contributing to memory decline and anxiety‐like behavior of the mice.

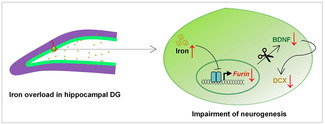

## 1 INTRODUCTION

The hippocampus is a critical brain area for all mammals responsible for learning and memory function, and it is also an important area that generates newborn neurons in the whole adult brain.^1^, ^2^, ^3^ Neural stem/progenitor cells (NSCs) in the subgranular zone (SGZ) of adult hippocampal dentate gyrus (DG) retain the capacity to generate new neurons throughout life, a process called adult hippocampal neurogenesis.^4^ There are a large number of NSCs in the SGZ region of adult hippocampus, which undergo self‐renewal, proliferation, and differentiation, and finally, give rise to newborn neurons and astroglia.^4^ The dendrites of the newborn neurons penetrate into the dendrite molecular layer of DG area, receiving signal input from the entorhinal cortex, and the axons interact with the neurons at CA3 subregion of hippocampus, thus integrating into the existing neuronal circuits.^5^

Accumulating evidence shows that hippocampal neurogenesis is abundant in healthy adult subjects and drops sharply in patients of Alzheimer's disease (AD).^6^, ^7^, ^8^ It has been implicated that the impairment of adult hippocampal neurogenesis is likely involved in the early stages of AD, which in turn exacerbates neuronal damage and mediates AD pathogenesis,^7^, ^8^ whereas promoting adult hippocampal neurogenesis ameliorates AD pathology and cognitive deficits.^9^, ^10^, ^11^ Therefore, investigation of the causes of impaired adult hippocampal neurogenesis may provide a good strategy for improving cognitive function by enhancing hippocampal neurogenesis in the treatment of AD.

Iron is one of the essential trace elements in human body, playing vital roles in various physiological activities, including synthesis of DNA and RNA, myelin, hemoglobin, and Fe‐S cluster biogenesis.^12^, ^13^, ^14^, ^15^ However, excessive iron can induce the production of reactive oxygen species (ROS) through Fenton reaction, thus participating in the pathogenesis of neurodegenerative diseases.^16^, ^17^ It has been well established that iron overload in hippocampus is tightly associated with the pathology of cognitive decline.^18^, ^19^, ^20^, ^21^ Modulation of brain iron metabolism or reducing iron content by iron chelators have shown great promises for alleviating AD symptoms.^22^, ^23^ Studies have also implicated that the abnormal brain iron metabolism appears in the early stage of AD,^19^, ^23^, ^24^, ^25^, ^26^ particularly the alteration of transferrin expression observed in the cortex and hippocampus of 3‐month‐old APP/PS1 transgenic mice,^25^ which is earlier than the occurrence of cognitive decline and the deposition of Aβ plaque. However, the roles and mechanisms of brain iron overload in the process of cognitive decline have not been fully elucidated. Whether iron overload in hippocampus directly impairs adult hippocampal neurogenesis, thus participating in the progress of cognitive decline, remains elusive.

In this study, we investigated the effects of the increased hippocampal iron on the proliferation and differentiation of NSCs in adult mice and further explored the underlying mechanisms of iron‐induced impairment of hippocampal neurogenesis. The findings in this study demonstrated a major regulatory role of elevated iron in the impairment of adult hippocampal neurogenesis, providing potential mechanisms for iron‐induced neurotoxicity and the causes of neurogenesis decline.

## 2 METHODS

### 2.1 Animals and treatments

The C57BL/6 mice were purchased from Huaxing Experimental Animal Center (Zhengzhou, China). All mice were housed in plastic cages on a 12‐h light/dark cycle and allowed free access to food and water. All procedures were carried out in accordance with the National Institutes of Health Guide for the Care and Use of Laboratory Animals and were approved by the Animal Ethics Committee of Hebei Normal University.

To establish the hippocampal iron overload mouse model, 1 μL of ferric ammonium citrate (FAC) at concentrations of 0, 0.5, 1, or 2 μg/μL was injected into bilateral hippocampi of 8‐week‐old C57BL/6 male mice under anesthesia using Hamilton micro syringe as previously reported.^21^ The infusion needle was stereotaxically implanted into hippocampus using the following coordinates relative to bregma: anteroposterior, −2 mm; lateral, +/−1.7 mm; and ventral, −1.6 mm (from dura). Mice were sacrificed after 1 or 2 weeks for analysis.

To detect the effects of FAC on differentiation of hippocampal NSCs, mice were intraperitoneally injected with bromodeoxyuridine (BrdU) at a dose of 100 mg/kg, twice a day for 4 consecutive days, and then FAC at 1 μg/μL was injected into bilateral hippocampi. Mice were sacrificed 1 week after FAC injection to assess BrdU‐positive and DCX‐positive cells in the hippocampal SGZ area, or mice were subjected to behavioral tests 3 weeks after FAC injection and sacrificed 4 weeks after FAC injection for immunostaining of BrdU‐positive and NeuN‐positive cells in the hippocampal DG.

### 2.2 Plasmid construction and transfection

The detailed information for furin overexpression plasmid (pAAV‐*Furin*) and control plasmid (pAAV‐*EGFP*) was described previously.^21^ The pAAV‐sh*Furin* and pAAV‐shControl plasmids were generated by cloning the reported *Furin* shRNA and scramble sequences into pAAV‐*EGFP* vector.^27^ For transfection, 1 μL of plasmid DNA at 1 μg/μL was injected into per hippocampus. Following injection, the brains were electroporated with square‐wave pulses from an Electro Square Porator ECM830 (BTX Inc) as described previously.^21^ The sh*Furin‐*injected mice and their controls were sacrificed 2 weeks after injection. The *Furin* overexpression mice were intrahippocampally injected with 1 μL FAC (1 μg/μL) after 1 week, while the vector control mice were injected with saline, FAC (1 μg/μL) or FAC (1 μg/μL) + deferoxamine (DFO, 0.2 μg/μL), and all the groups were sacrificed for analysis after another week.

### 2.3 Behavioral tests

The Morris water maze was used to detect the spatial learning and memory performances of mice. The apparatus consisted of a circular tank (diameter = 120 cm and height = 50 cm) filled with water that was divided into four quadrants. During the training stage, mice were trained to identify the platform (diameter = 9 cm) above the water based on the cues on the wall and then tested for 4 consecutive days to record the times used to find the hidden platform (1 cm under the water). Each trail was up to 120 s, and the escape latency time was recorded as 120 s if the mouse did not find the platform. Then, a probe test was performed after removing the platform, in which the percentage of time that mouse spent searching the platform in the target quadrant in 120 s was recorded, as well as the number of times that mouse crossed the former location of the platform.

The open‐field test was used to determine the anxiety‐like behavior of mice. The apparatus consisted of a square box (43 × 43 cm), and the test area was divided into center (15 × 15 cm) and peripheral zones. Each mouse was placed in the central area and was allowed to explore for 10 min. The time, distance, and resting time that mouse spent in the center and the total were recorded and analyzed by an automatic video‐tracking system. Before switching animals, the field was wiped thoroughly with alcohol to avoid the cue smell.

### 2.4 Western blot

Dissected hippocampal tissues were homogenized in RIPA buffer (1% NP‐40, 0.1% SDS, 0.5% PMSF, and 4% Roche protease inhibitor) by sonication. After centrifugating at 12,000*g* for 20 min at 4°C, the supernatant was collected and protein concentration was measured. A total cell extract containing ~30 μg of protein was subjected to SDS‐PAGE and then transferred onto NC membrane. The membrane was blocked by incubation with 5% skim milk for 90 min and then hybridized overnight with primary antibodies at 4°C. The primary antibodies used include anti‐ferritin light chain (ab109373, Abcam), anti‐ferritin heavy chain (ab183781, Abcam), anti‐TfR1 (13–6800, Invitrogen), anti‐Bcl2 (12789‐1‐AP, Proteintech), anti‐Bax (50599‐2‐Ig, Proteintech), anti‐brain‐derived neurotrophic factor (BDNF) (ab108319, Abcam), anti‐furin (ab183495, Abcam), and anti‐β‐actin (CW0096, CWBIO). After washing with tris‐buffer saline with 0.05% Tween‐20, the membrane was incubated at room temperature for 90 min with anti‐rabbit or anti‐mouse secondary antibody conjugated with horseradish peroxide (Zhongshan Biotechnology). Immunoreactive bands were detected by the enhanced chemiluminescence detection kit (Amersham, UK) and visualized by a Bio‐Rad chemiluminescence imager.

### 2.5 Immunofluorescence staining

The mouse brains were fixed with 4% paraformaldehyde in 0.1 M phosphate buffer overnight and then equilibrated in 30% sucrose for 48 h. Serial coronal sections were cut at 20 μm thick on a freezing microtome (Leica CM1950), mounted onto slides, and stored at −80°C until use. The sections were washed with 0.01 M PBS and incubated for 10 min in 3% hydrogen peroxide (H_2_O_2_) to inhibit the activity of endogenous peroxidase. For antigen recovery, the sections were microwaved in 10 mM citrate buffer for 10 min, cooled to room temperature, and washed with PBS. After blocking with goat serum for 1 h, the primary antibody was added and incubated overnight at 4°C. Primary antibodies include anti‐Ki67 (ab15580, Abcam), anti‐DCX (#4604, CST), BrdU (ab6326, Abcam), anti‐NeuN (ab104224, Abcam), anti‐cleaved Caspase‐3 (#9661, CST), anti‐BDNF (ab108319, Abcam), and anti‐furin (ab183495, Abcam). For BrdU staining, before blocking, the slices were treated with 1 M HCl for 30 min on ice, 2 M HCl for 10 min at room temperature, and 20 min at 37°C, and then neutralized with 0.1 M borate buffer for 10 min at room temperature. The sections were washed with PBS, and incubated with RHODAMINE‐conjugated or FITC‐conjugated secondary antibodies at 37°C for 1 h. Nuclei were counterstained with DAPI. Finally, after washing and mounting, the sections were photographed under a fluorescence confocal microscope (Olympus FV3000).

For qualitative analysis, three or more sections per mouse from similar stereotaxic positions were stained. For Ki67, DCX, BrdU, NeuN, and cleaved Caspase‐3 immunostaining, the numbers of positive cells in hippocampal DG region were quantified. The results of Ki67‐, DCX‐, BrdU‐, and NeuN‐positive cells were presented as the average numbers of positive cells in a cubic volume (mm^3^) of DG.^28^ The results of cleaved caspase‐3‐positive cells were presented as the percentage of the total DAPI‐stained cells in hippocampal DG of different mice. For BDNF and furin immunostaining, the relative fluorescence intensity in DG area was quantified and normalized to the fold changes in the control group.

### 2.6 Quantitative reverse transcription polymerase chain reaction (qRT‐PCR)

The total RNA from hippocampus was extracted by TRIzol reagent (Invitrogen) and reverse‐transcribed with MMLV reverse transcriptase and Oligo‐dT primers (TaKaRa) into cDNA. The qRT‐PCR was performed with an UltraSYBR mixture (CW0957, CWBIO) according to the manufacturer's instructions. The primers used are listed in Table S1. The dissolution curve was analyzed for each reaction to confirm the specificity of the primers. The *GAPDH* gene was used as the internal reference. The cycle time (Ct) value for the gene of interest in each animal was firstly normalized by its own β‐actin (ΔCt), and then the relative differences between the control and the interest groups were calculated using the equation 2^−ΔΔCt^, and expressed as relative fold changes of the control group.

### 2.7 Perl's staining

As described previously,^29^ brain sections were washed with 0.01 M PBS, and then incubated with 3% H_2_O_2_ for 20 min at room temperature. After washing, the ready‐to‐use 1% Perl's dye solution (2% ferrocyanide and 2% hydrochloric acid in equal volumes) was added, and the sections were incubated for 6 h. After washing, color development was enhanced by treating with 3, 3′‐diaminobenzidine tetrahydrochloride‐hydrogen peroxide and stopped by rinsing with distilled water for 30 min. The sections were then dehydrated with gradient alcohols, transparentized by xylene, and finally, sealed with neutral gum and examined under a light microscope.

### 2.8 TUNEL staining

The apoptotic cells in the sections were assessed by a TUNEL BrightGreen Apoptosis Detection Kit (Vazyme) by following the manufacturer's protocol. Nuclei were counterstained with DAPI. All sections were visualized and photographed under a fluorescence confocal microscope. The numbers of TUNEL‐positive cells located at hippocampal DG in different mice were quantified and expressed as the percentage of TUNEL‐positive cells relative to the total cells (DAPI‐labeled nuclei).

### 2.9 Statistical analysis

Data were presented as means ± standard error of mean (SEM). Data sets were tested for normality distribution using the Shapiro–Wilk test, and those that passed the normality test were proceeded for statistical analysis. The statistical analyses in multiple groups were performed by one‐way analysis of variance with Tukey's post hoc tests using SPSS 21.0 software. The comparisons between two groups were performed using Student *t*‐tests. The *p* values of <0.05 were considered to be statistically significant differences.

## 3 RESULTS

### 3.1 Intrahippocampal iron injection impaired adult hippocampal neurogenesis in a dose‐dependent manner

**FIGURE 1:**
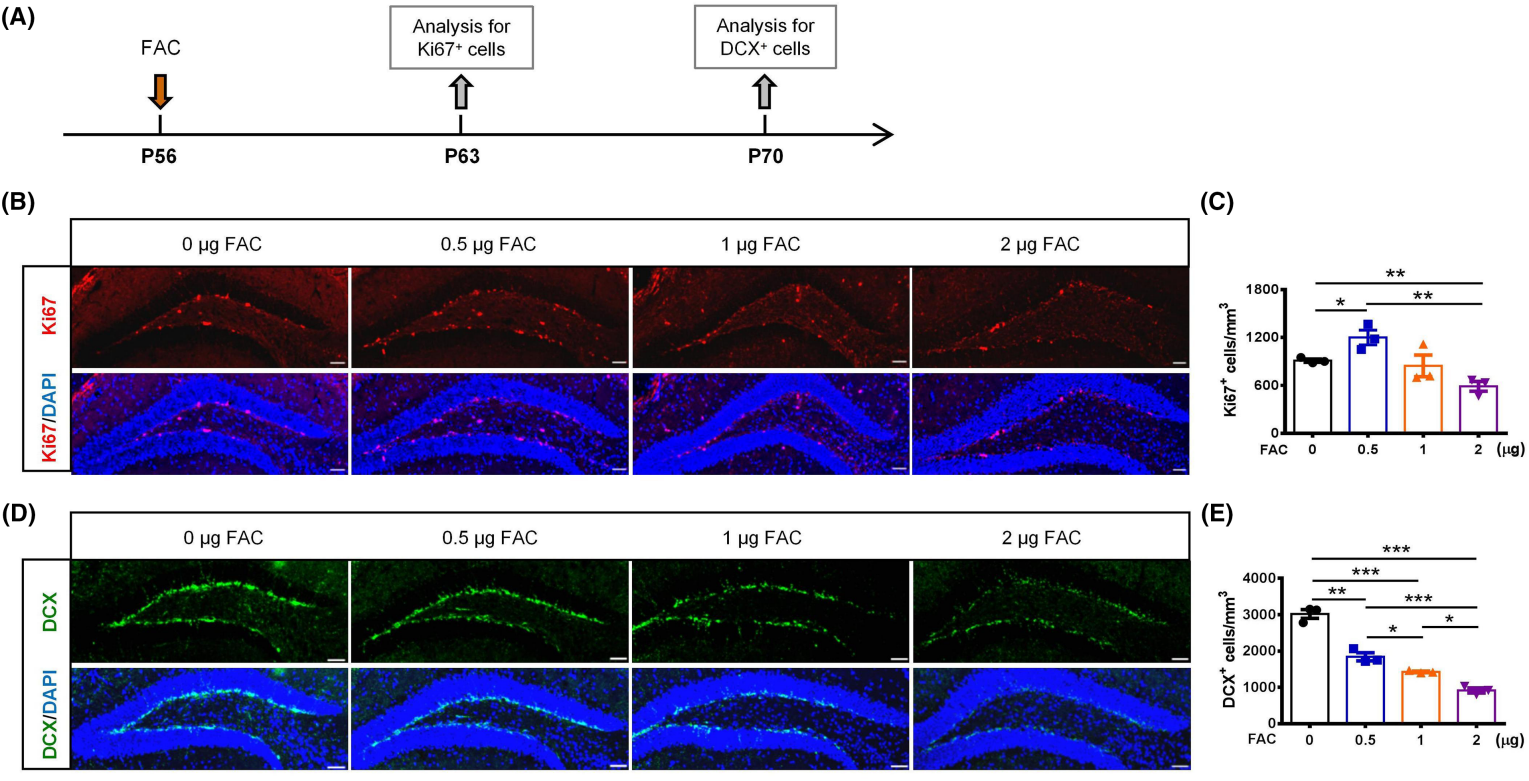
Hippocampal iron overload impaired neurogenesis dose dependently. (A) Experimental scheme for ferric ammonium citrate (FAC) injection and sample analyses. (B) and(C) Immunostaining images of Ki67^+^ cells (B) and quantification (C) in the hippocampal dentate gyrus (DG) of different FAC‐injected mice. Scale bar = 50 μm. (D) and(E) Immunostaining images of DCX^+^ cells (D) and quantification (E) in the hippocampal DG of different FAC‐injected mice. Scale bar = 50 μM. *p < 0.05, **p < 0.01, and ***p < 0.001 (mean ± SEM, *n* = 3 per group).

To investigate whether elevated iron content in hippocampus impaired hippocampal neurogenesis, different doses of FAC were injected into the bilateral hippocampi of 8‐week‐old mice (P56). Brains were obtained 7 or 14 days post‐injection for detecting cells in SGZ undergoing proliferation or differentiation, respectively (Figure 1A). The immunofluorescence staining results showed that the number of Ki67^+^‐proliferating cells in hippocampal DG had a significant increase in 0.5 μg FAC‐treated mice, but decreased substantially in 2 μg FAC‐treated mice, while no significant change was observed in the 1 μg FAC‐treated mice compared to the control mice (Figure 1B,C). On the other hand, the DCX^+^ neuroblasts in hippocampal SGZ of 0.5, 1, and 2 μg FAC‐injected mice were all significantly decreased, notably in a dose‐dependent manner, compared with the control mice (Figure 1D,E). These results suggested that the elevated iron content in hippocampus impaired adult hippocampal neurogenesis, and the impact may be closely associated with the destruction of differentiation process of NSCs by iron overload.

### 3.2 Iron overload during the differentiation of NSCs reduced the number of newborn immature neurons

**FIGURE 2:**
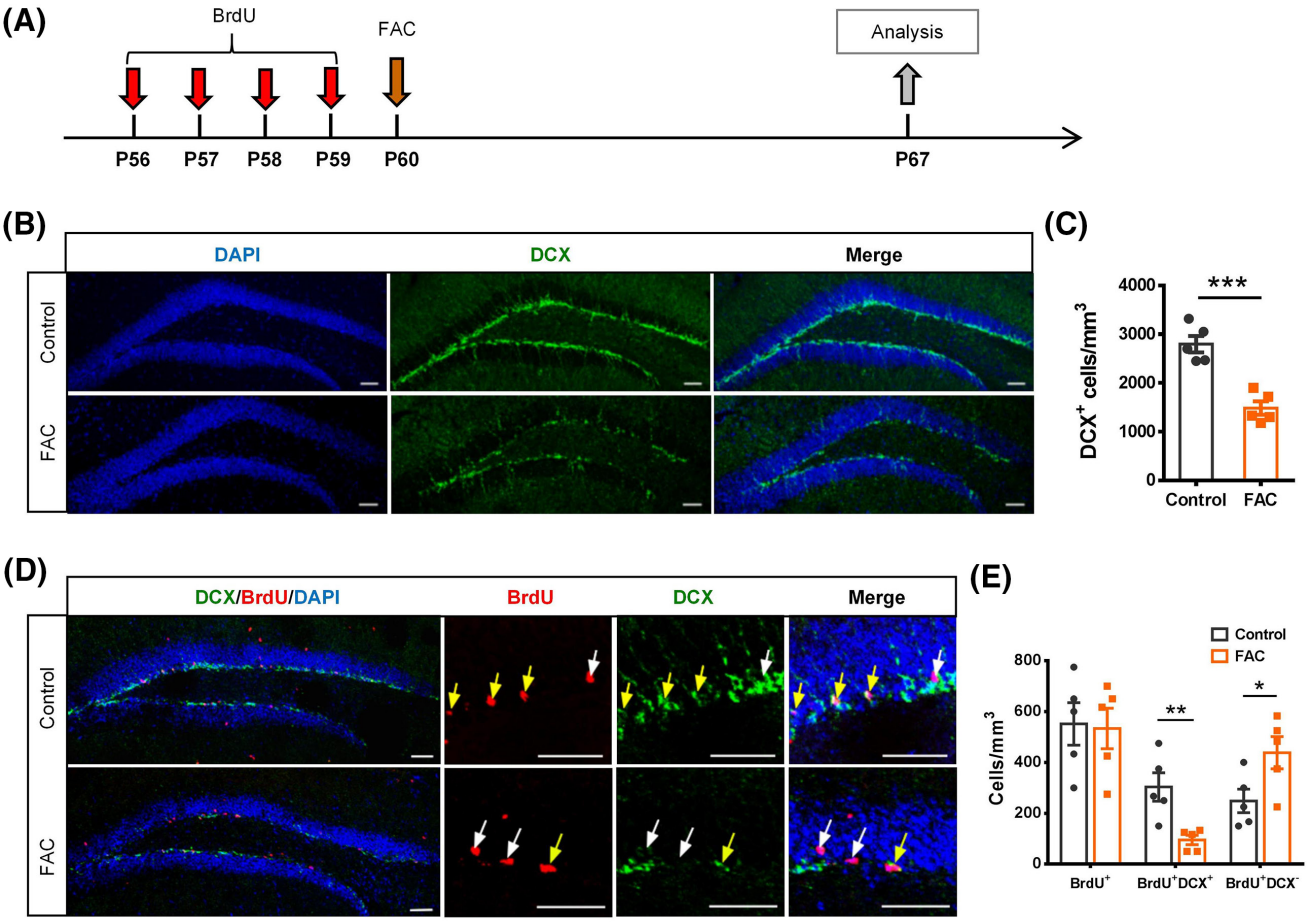
Hippocampal iron overload during the differentiation of neural stem cells reduced the number of newborn immature neurons. (A) Experimental scheme for BrdU pulse labeling, ferric ammonium citrate (FAC) treatment, and sample analysis. (B) and (C) Immunostaining images of DCX^+^ cells (B) and quantification (C) in the hippocampal dentate gyrus of FAC‐injected and control mice. Scale bar = 50 μm. (D) and (E) Immunostaining images of BrdU^+^ and DCX^+^ cells (D), and quantifications of BrdU^+^ cells, BrdU^+^DCX^+^ cells, and BrdU^+^DCX^−^ cells (E). Scale bar = 50 μm. *p < 0.05, **p < 0.01, and ***p < 0.001 (mean ± SEM, *n* = 5 per group).

To explore the impact of elevated iron content on the differentiation of hippocampal NSCs, P56 mice were intraperitoneally injected with BrdU for 4 days to label the proliferating cells, and then 1 μg FAC was injected intrahippocampally at P60 to influence the differentiation of the BrdU‐labeled proliferative stem cells.^28^ Mice were sacrificed for analysis 1 week after FAC injection (Figure 2A). Immunofluorescence staining results showed that the DCX^+^ neuroblasts in the SGZ of FAC‐treated mice decreased significantly, compared to that of the controls (Figure 2B,C). Particularly, the number of newborn immature DCX^+^BrdU^+^ neurons had a remarkable reduction in FAC‐treated mice (Figure 2D,E). Notably, the total number of BrdU^+^ cells was not decreased in the FAC group, whereas the BrdU^+^DCX^−^ cells (pointed by white arrows) increased significantly (Figure 2D,E). These suggested that iron treatment severely affected the differentiation of BrdU‐labeled stem cells toward neurons.

### 3.3 Iron overload decreased the number of newborn neurons and impaired cognition

**FIGURE 3:**
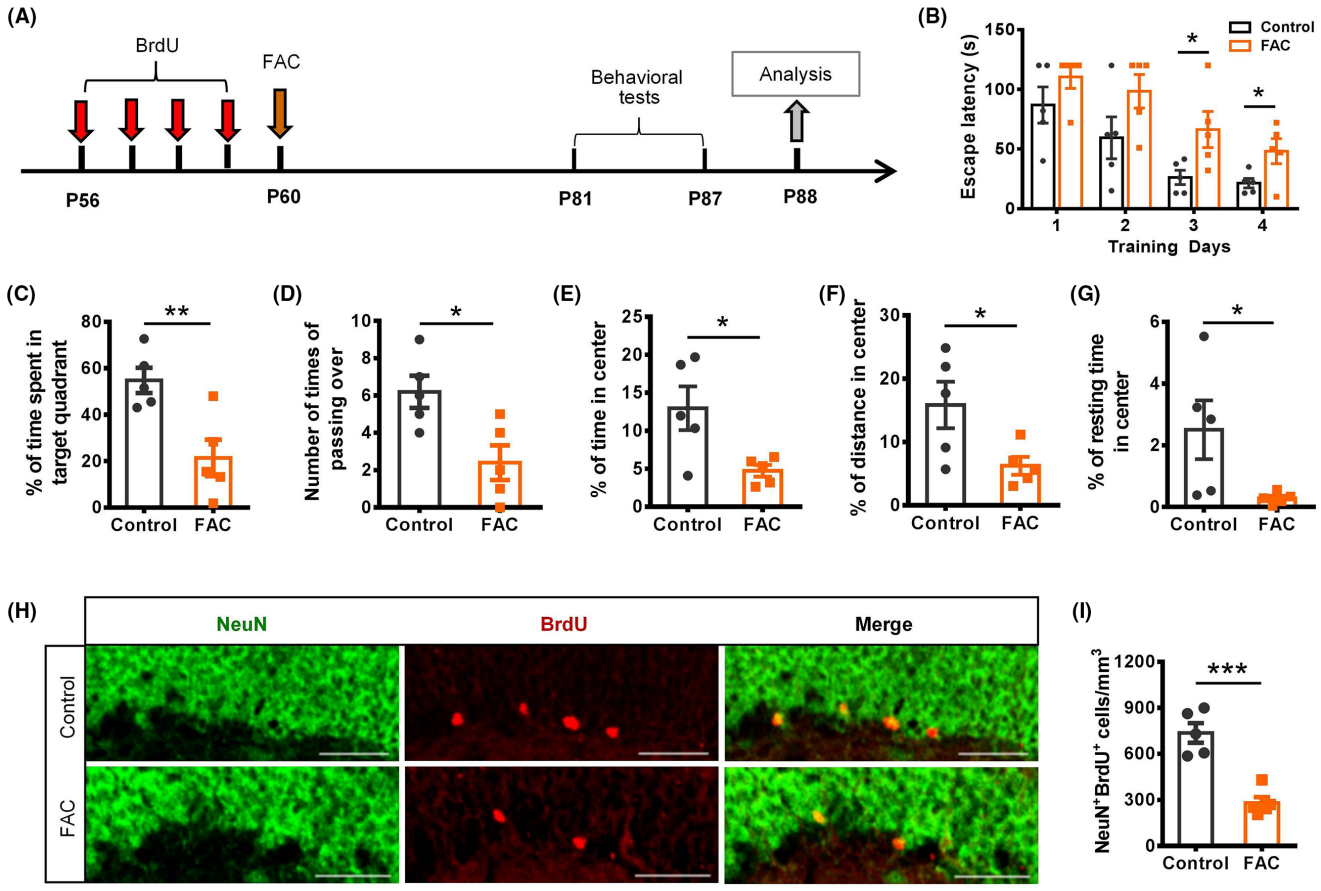
Iron overload impaired cognition of mice and decreased the number of newborn neurons. (A) Experimental scheme for BrdU pulse labeling, ferric ammonium citrate (FAC) treatment, behavioral tests, and sample analysis. (B)–(D) The escape latency time during the training stage (B), the percentage of time spent in target quadrant (C), and number of times of crossing over the former platform area (D) during the probe stage in the water maze tests. (E)–(G) The percentage of time (E), distance (F), and resting time (G) spent in the zone of center in the open‐field tests. (H) and (I) Immunostaining images of BrdU^+^ and NeuN^+^ cells (H), and quantification (I) in the hippocampal dentate gyrus of different mice. Scale bar = 50 μm. *p < 0.05, **p < 0.01, and ***p < 0.001 (mean ± SEM, *n* = 5 per group).

To explore the impact of elevated iron on the cognition of FAC‐injected mice, behavioral tests were performed 3 weeks after FAC injection (Figure 3A). The results showed that FAC‐treated mice had declined learning and memory abilities in water maze tests, as shown by the longer escape time to find the platform during the training stage (Figure 3B), and less time spent in the target quadrant and passing over the former platform area during the probe stage (Figure 3C,D). Besides, the FAC‐treated mice also showed anxiety‐like behavior as indicated in the open‐field tests. The percentage of time, distance, and resting time spent in the center field of FAC‐treated mice were substantially decreased (Figure 3E–G). Furthermore, consistent with the behavioral performance, immunostaining results showed that the newborn neurons (NeuN^+^BrdU^+^) in hippocampal DG of FAC‐treated mice were decreased substantially (Figure 3H,I). These suggested that the elevated iron content in hippocampus caused cognitive impairment in mice, which is likely linked to the decline in hippocampal neurogenesis.

### 3.4 Elevated iron in hippocampus did not lead to increased apoptosis in hippocampal DG

**FIGURE 4:**
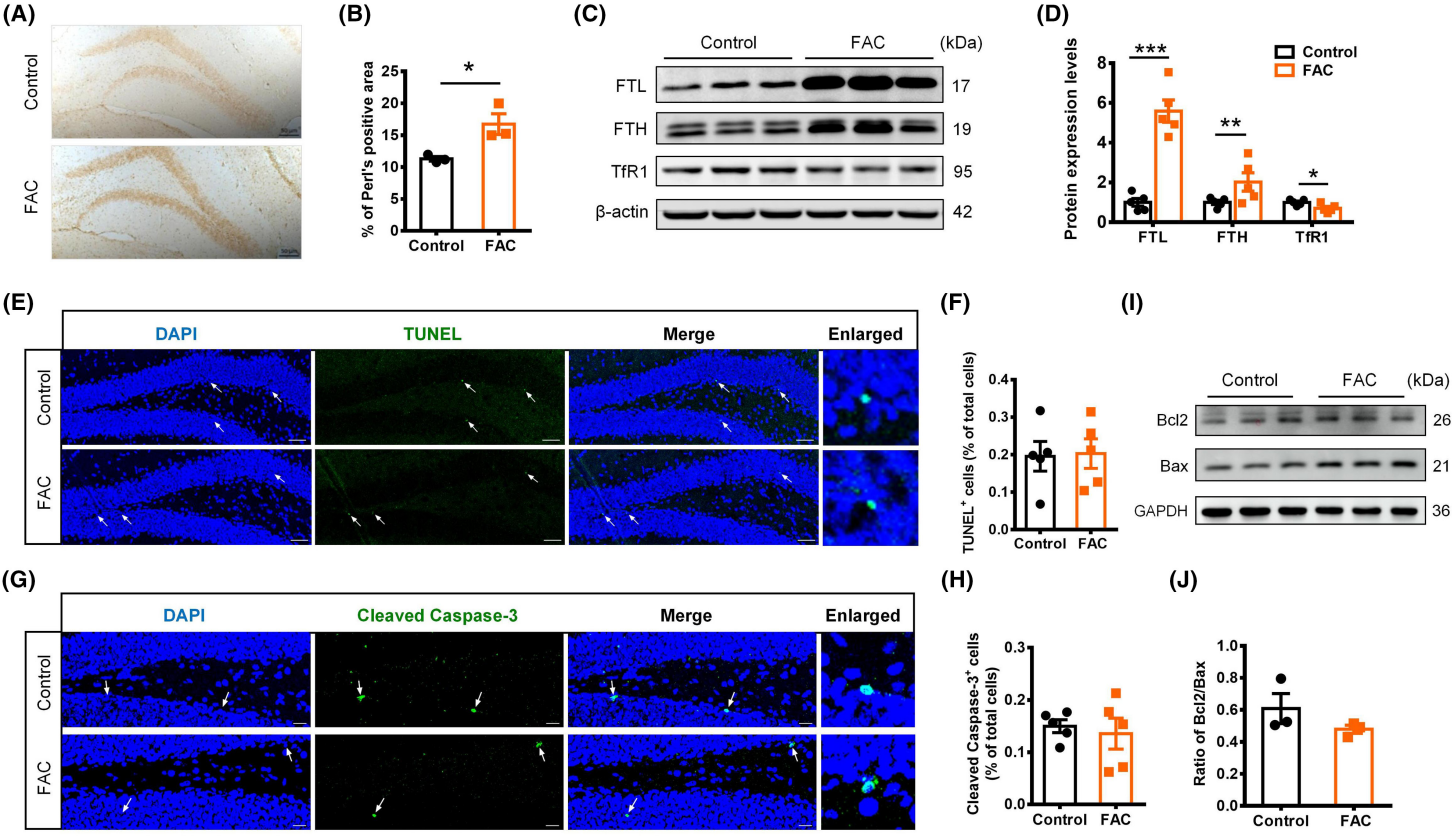
Elevated iron level did not increase the apoptosis in hippocampal dentate gyrus (DG). (A) and (B) Perl's staining images (A) and quantification (B) in hippocampal DG of ferric ammonium citrate‐injected and control mice (scale bar = 50 μm, *n* = 3 per group). (C) and (D) Western blot images (C) and quantifications (D) of FTL, FTH, and TfR1 in the hippocampus of different mice (*n* = 5 per group). (E) and (F) TUNEL staining for apoptotic cells in hippocampal DG of different mice (E, scale bar = 50 μm) and the quantification analysis (F). (G) and (H) Immunostaining images of cleaved caspase‐3‐positive cells in hippocampal DG of different mice (G, scale bar = 20 μm) and the quantification analysis (H). (I) and (J) Western blot images of Bcl2 and Bax expression (I) and quantification of Bcl2/Bax ratios (J) in the hippocampus of different mice (*n* = 3 per group). Data are expressed as mean ± SEM. *p < 0.05, **p < 0.01, and ***p < 0.001.

To investigate whether the elevated iron level increased the apoptosis of developing NSCs and hence decreased the number of newborn neurons, we determined the levels of iron and apoptotic cells in hippocampus of different mice. The increase in iron content in hippocampal DG region was confirmed by Perl's stain (Figure 4A,B). Consistently, western blot also showed an increased expression of iron storage protein ferritin, including ferritin light chain (FTL) and ferritin heavy chain (FTL), and a decreased expression of iron uptake protein transferrin receptor 1 (TfR1) (Figure 4C,D). These changes reflect an increase in cellular iron content and its feedback regulation on expression of iron storage and uptake molecules in hippocampus.^30^

The apoptosis in hippocampal DG region was then detected by TUNEL staining. Compared with the control mice, the number of apoptotic cells in DG area of FAC‐treated mice did not show any increase (Figure 4E,F). Consistent with that, immunofluorescence staining showed that the cleaved caspase‐3‐positive cells in the hippocampal DG of FAC‐treated mice did not increase either (Figure 4G,H). Additionally, the protein expression ratio of Bcl2 to Bax had no statistically significant difference between FAC‐treated mice and control mice (Figure 4I,J). These observations suggested that iron injection at the current dose did not trigger a significant activation of apoptosis in hippocampal DG. This was also consistent with the observation that no significant decrease in total BrdU^+^ cells was detected in the hippocampal DG of FAC‐treated mice, indicating that the impairment of neurogenesis induced by iron was not likely result from the apoptosis of developing NSCs.

### 3.5 Iron overload suppressed furin expression and downregulated brain‐derived neurotrophic factor in hippocampus

**FIGURE 5:**
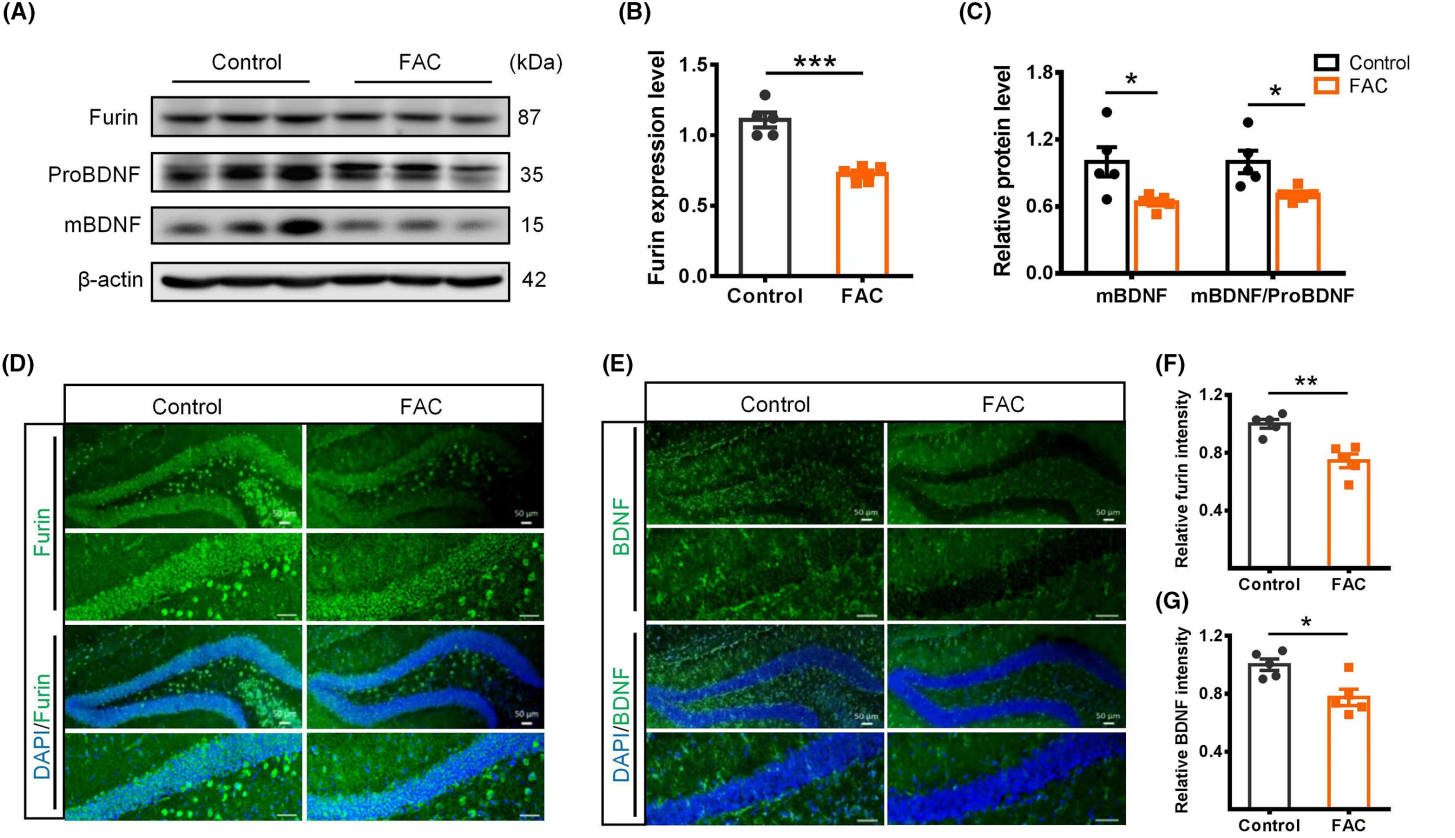
Iron overload suppressed furin expression and down‐regulated brain‐derived neurotrophic factor (BDNF) in hippocampus. (A)–(C) Western blot images of furin, proBDNF, and mBDNF (A), and quantifications of furin expression (B), mBDNF, and mBDNF/proBDNF levels (C) in hippocampus of different mice. (D)–(G) Immunostaining images of furin (D) and BDNF (E) and their quantification analyses (F) and (G) in the hippocampal dentate gyrus of different mice (scale bar = 50 μm). Data are expressed as mean ± SEM (*n* = 5 per group). *p < 0.05, **p < 0.01, and ***p < 0.001.

It has been reported that the expression of proprotein convertase furin was downregulated by iron overload,^21^, ^31^, ^32^ which subsequently reduced the maturation of BDNF.^21^ BDNF is a neurotrophic factor, which is highly expressed in hippocampus and plays a crucial role in promoting the growth, differentiation, and maturation of NSCs.^33^, ^34^, ^35^ We therefore determined the expression of furin and BDNF in the hippocampus of FAC‐treated and control mice. Western blot showed that the expression of furin protein (Figure 5A,B) and mRNA (Figure S1) were both decreased significantly in the hippocampus of FAC‐treated mice. The mature BDNF (mBDNF) level and BDNF mRNA expression were also decreased (Figure 5A,C; Figure S1). The reductions in furin and BDNF in DG areas of FAC‐treated mice were also confirmed by immunofluorescence staining (Figure 5D–G). Furthermore, the expression of BDNF receptor *TrKb* and intracellular signaling molecule *CamK2b* were also downregulated in FAC‐treated mice (Figure S1), indicating a reduced BDNF signaling caused by iron overload. These inferred that the reduction in furin and mBDNF induced by iron treatment might account for the decline in hippocampal neurogenesis.

### 3.6 Modulating furin expression in hippocampus altered hippocampal neurogenesis

**FIGURE 6:**
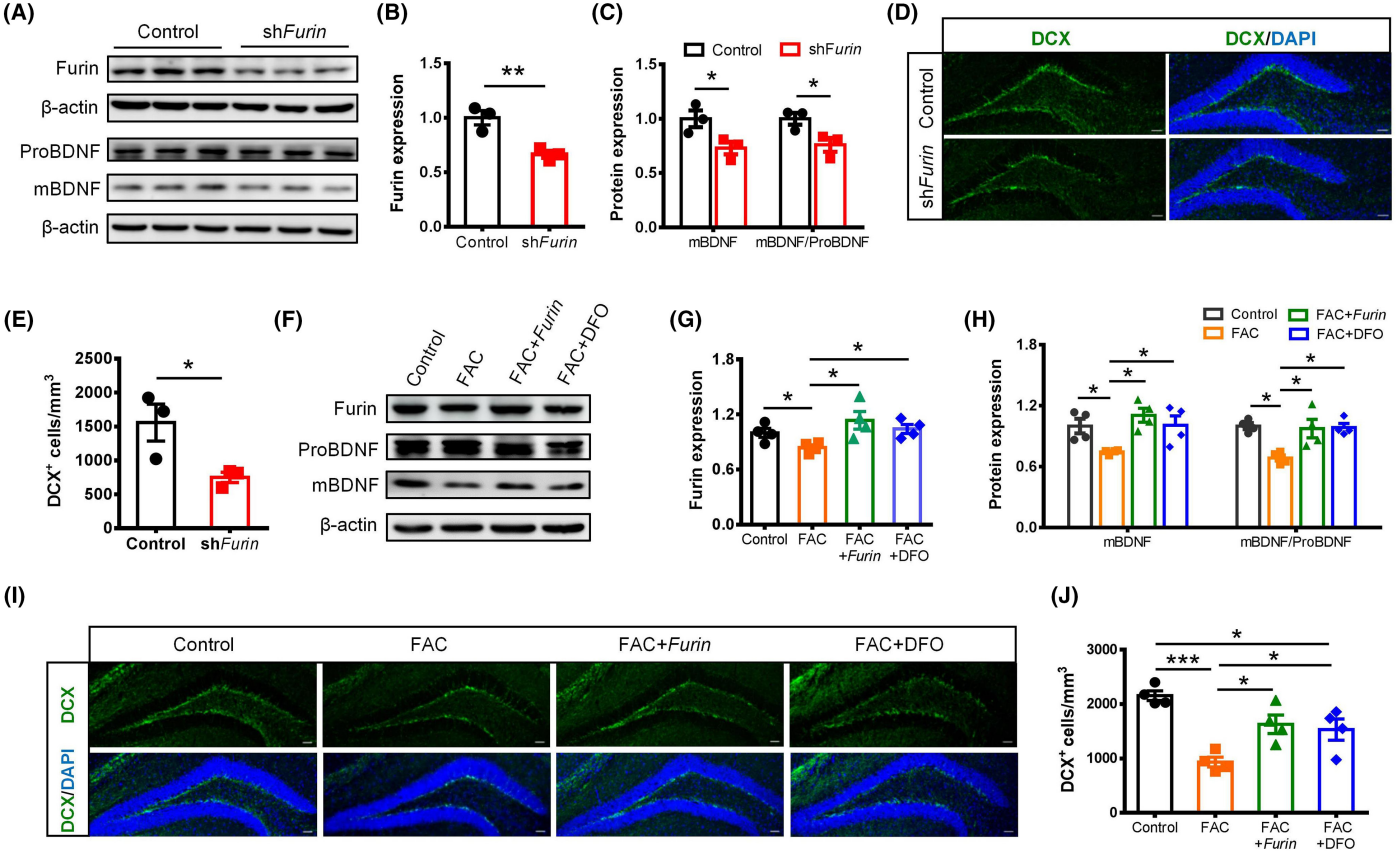
Modulation of furin expression in hippocampus affected hippocampal neurogenesis. (A)–(C) Western blot images (A) and quantification of furin (B) and brain‐derived neurotrophic factor (BDNF) expression (C) in hippocampus of sh*Furin* and control mice (*n* = 3 per group). (D) and (E) Immunostaining images of DCX^+^ cells (D) and quantification of cell numbers (E) in the hippocampal dentate gyrus (DG) of sh*Furin* and control mice (scale bar = 50 μm, *n* = 3). (F)–(H) Western blot images (F) and quantification of furin (G) and BDNF (H) expression in hippocampus of *Furin* overexpression and other mice (*n* = 4 per group). (I) and (J) Immunostaining images of DCX^+^ cells (I) and quantification of cell numbers (J) in the hippocampal DG of *Furin* overexpression and other mice (scale bar = 50 μm, *n* = 4). Data are expressed as mean ± SEM. *p < 0.05, **p < 0.01, and ***p < 0.001.

To investigate if reducing furin expression damaged hippocampal neurogenesis, sh*Furin* and control scramble sequences were constructed into pAAV vector and injected into 8‐week‐old mice. Results showed that furin expression in hippocampus was significantly downregulated in the sh*Furin* group at 2 weeks post‐injection (Figure 6A,B), and the mBDNF level was also reduced (Figure 6A,C). Meantime, the DCX‐positive cells in the hippocampal DG region of sh*Furin* mice were markedly decreased compared with that of the control mice (Figure 6D,E). In contrast, *Furin* was overexpressed in the hippocampus followed by FAC injection after 1 week (FAC + *Furin* group). For comparison, mice injected with control pAAV + PBS (control group), control pAAV + FAC (FAC group), or control pAAV + FAC and DFO (FAC + DFD group) were used as controls (Figure S2A). The results showed that furin has an increased expression in the FAC + *Furin* and FAC + DFO groups (Figure 6F,G; Figure S2B,C). Similarly, the levels of mBDNF were also upregulated in the FAC + *Furin* and FAC + DFO groups (Figure 6F,H). Meanwhile, the numbers of DCX‐positive cells in the hippocampal DG region of the FAC + *Furin* mice and FAC + DFO mice were largely increased compared with the FAC group (Figure 6I,J). These suggested that overexpression of furin protected mice from iron overload‐induced impairment of hippocampal neurogenesis.

## 4 DISCUSSION

Iron is a pivotal trace element for maintaining the physiological function of cells. Accumulating evidence has suggested that iron deficiency in the brain links to neurological developmental disorders with impairment of language acquisition, physical activity, and cognition,^36^, ^37^ while iron overload in the brain is closely associated with pathophysiological conditions including cognitive decline, motor dysfunctions, and psychiatric disorders.^19^, ^38^, ^39^ Brain iron overload has been demonstrated to cause excess ROS production, which leads to oxidative stress, inflammation, and mitochondrial dysfunction, consequently resulting in apoptosis, ferroptosis, or other forms of neuronal death.^40^, ^41^, ^42^ However, whether iron overload in the brain affects the generation of newborn neurons during adult hippocampal neurogenesis, and thus contributes to the pathogenesis of neurological diseases, has not been reported.

In this study, by establishing hippocampal iron‐overloaded mouse models, we investigated the effect of iron overload on adult hippocampal neurogenesis. As increasing iron contents in hippocampus, the proliferating Ki67‐positive cells in the DG area of hippocampus increased at low dose of iron but then decreased significantly at high dose of iron. Interestingly, the DCX‐positive cells, representing the newborn neurons, decreased dose dependently with the increase in iron. These inferred that iron overload in hippocampus was harmful to neurogenesis. Furthermore, in BrdU pulse labeling experiment, the inhibitory effect of iron overload on differentiation of hippocampal NSCs was confirmed by the significantly decreased newborn BrdU^+^DCX^+^ cells. Consistently, the newborn mature neurons (BrdU^+^NeuN^+^ cells) in the hippocampus of iron‐injected mice were reduced significantly as well. It was noted that the current iron dosage did not cause severe apoptosis in the hippocampal DG, which was consistent with the observation that the total number of BrdU^+^ cells had not significantly decreased. In association with the behavioral performances of different mice, it may suggest that the cognitive decline and anxiety‐like emotion that we observed in FAC‐treated mice possibly attributed in part to the damage effect of iron on hippocampal neurogenesis.

BDNF, as one of the most important neurotrophic factors in brain, is closely related to the pathophysiological process of many neurological diseases.^43^, ^44^, ^45^ Moreover, abundant evidence has shown that the level of BDNF in the hippocampus also directly affects adult hippocampal neurogenesis.^9^, ^31^, ^34^ Furthermore, the regulation of iron on BDNF level has been demonstrated previously.^46^, ^47^ Consistently, our results revealed a significant decrease in BDNF expression, especially the mBDNF level, in the brain of iron‐treated mice. These suggested that the damaging effect of iron overload on hippocampal neurogenesis may involve the participation of BDNF downregulation.

Furin, the first proprotein convertase found in mammals,^48^ is the major enzyme for the proteolysis of proBDNF to generate mBDNF.^49^ The aberrant activity of furin leads to a significant change in BDNF maturation,^49^, ^50^ which is closely associated with AD pathogenesis.^51^, ^52^, ^53^ The emerging role of furin in neurodegenerative and neuropsychiatric diseases has been elucidated.^54^, ^55^ The expression levels of furin in the cortex of AD patients and model mice are reduced.^56^, ^57^ The impact of iron concentration on the level of furin expression has been reported. Iron overload decreases furin expression,^31^, ^32^ while iron deficiency increases furin expression.^31^ Our recent work has identified furin as a bridging factor between iron overload and cognitive decline.^21^ Iron overload in hippocampus significantly suppressed furin expression at the transcription level, while administration of iron chelator could upregulate furin expression and rescue iron‐induced synaptic damage and memory decline in mice.^21^ Therefore, we proposed that the reduction in furin and its influence on BDNF maturation may account for iron‐induced downregulation of hippocampal neurogenesis. We then modulated furin expression in the hippocampus and testified its impact on FAC‐induced damage of hippocampal neurogenesis. The results showed that knockdown of furin expression in hippocampus caused the reduction in DCX^+^ cells, while overexpression of furin partially rescued FAC‐induced damage of neurogenesis. These inferred that iron–furin–BDNF signaling pathway could be one of the causes for neurogenesis impairment.

Taken together, this study demonstrated that hippocampal iron overload impaired adult hippocampal neurogenesis, which was closely associated with iron‐induced decreases in furin expression and BDNF maturation. Increasing furin expression or chelating iron by DFO could partially restore the level of adult hippocampal neurogenesis in iron‐overloaded mice. Our study provides new insights into the molecular mechanisms of iron‐induced cognitive dysfunction and the causes of neurogenesis injury. It also renders modulating iron homeostasis and furin expression as potential therapeutic strategies to enhance hippocampal neurogenesis for treatment of iron overload‐related neurodegenerative diseases.

## CONFLICT OF INTEREST STATEMENT

The authors declare that there is no potential conflict of interest.

## CONSENT TO PARTICIPATE

Not applicable.

## CONSENT TO PUBLISH

Not applicable.

## Supporting information


Data S1.

## Data Availability

Additional data can be found in the Supplemental Material. No datasets were generated in the current study. Materials in this study are available from the corresponding authors upon reasonable request.
