# Supplementary material for: Iron overload suppresses hippocampal neurogenesis in adult mice: Implication for iron dysregulation‐linked neurological diseases
Source: CNS Neurosci Ther. 2023 Aug 7;30(2):e14394. doi: 10.1111/cns.14394 (PMC10848078; doi:10.1111/cns.14394)
Supplement: Supplementary file 1 — Data S1. [file CNS-30-e14394-s001.pdf]

Supplementary tables and figures

Table S1. Primer sequences used for qRT-PCR.

Genes	Sequences (5'-3')
Furin	Forward: GCGAGACCTGAATGTGAAGGA<br>Reverse: CATAATTGCCTGCTAGGTCGG
BDNF	Forward: CTGTATCAAAAGGCCAACTGAA<br>Reverse: GTGTCTATCCTTATGAATCGCCA
Trkb	Forward: TGACGCAGTCGCAGATGCTG<br>Reverse: TTTCCTGTACATGATGCTCTCTGG
Camk2b	Forward: TAGAGGATGAAGATGCCAAAGC<br>Reverse: TCAGGCTCAAATGAGGTCAGG
GAPDH	Forward: TGACTTCAACAGCGACACCCA<br>Reverse: CACCCTGTTGCTGTAGCCAAA

Figure S1

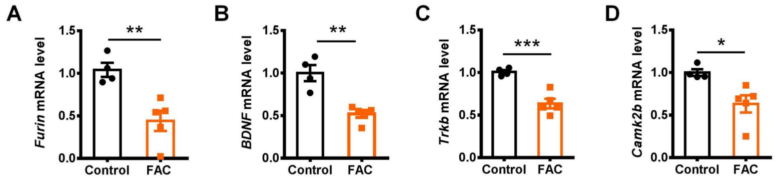

Fig. S1. The mRNAs of *Furin*, *BDNF*, *Trkb* and *Camk2b* were decreased. The qRT-PCR results showing the relative mRNA levels of *Furin* (A), *BDNF* (B), *Trkb* (C) and *Camk2b* (D) in control and FAC-treated mice. n = 4, 5 in the control and FAC group. *p < 0.05, **p < 0.01 and ***p < 0.001.

Figure S2

A

Groups	Injection 1	Injection 2
Control	pAAV control	Saline
FAC	pAAV control	FAC
FAC+ *Furin*	pAAV-*Furin*	FAC
FAC+ DFO	pAAV control	FAC+DFO

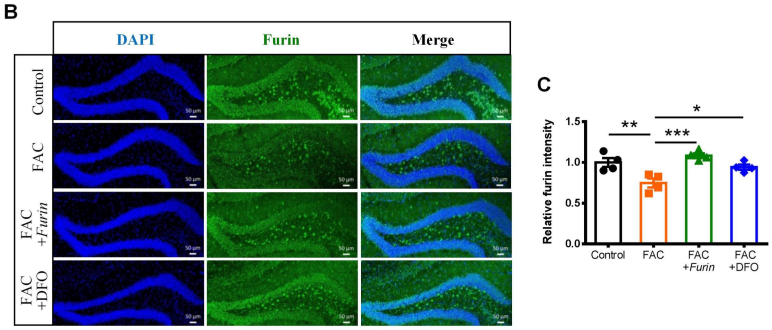

Fig. S2. Injection formulas and *Furin* expression levels. (A) The injection formulas for the *Furin* overexpression and control groups. (B-C) Immunostaining images (B) and the relative intensity (C) of furin in the hippocampal DG of different groups of mice (scale bar = 50 μm, n = 4). Data are expressed as mean ± SEM. *$p < 0.05$, **$p < 0.01$ and ***$p < 0.001$.
